# Combined enriched environment and fluoxetine enhance myelin protein expression in the prefrontal cortex of a chronic unpredictable stress depression model

**DOI:** 10.1186/s12993-025-00282-1

**Published:** 2025-06-11

**Authors:** Jingyang Gu, Cong Liu, Yan Li, Laipeng Feng, Mengjun Geng, Jiao Dong, Jinhong Han, Liqin Zhao, Qiujing Shao, Hui-Ying Wang, Chang-Hong Wang

**Affiliations:** 1 Department of Psychiatry, The Second Affiliated Hospital of Xinxiang Medical University, Xinxiang, Henan, 453002 China; 2 Henan Collaborative Innovation Center of Prevention and Treatment of Mental Disorder, The Second Affiliated Hospital of Xinxiang Medical University, 453002 Xinxiang, Henan, China; 3 Zhengzhou University, Zhengzhou, Henan, 450000 China; 4 Henan Key Lab of Biological Psychiatry, Xinxiang Medical University, Xinxiang, Henan 453002 China; 5 Brain Institute, Henan Academy of Innovations in Medical Science, Zhengzhou, Henan, 451163 China; 6 Department of Basic Medical, Xinxiang Medical University, Xinxiang, Henan, 453003 China

**Keywords:** Enriched environment, Fluoxetine, Myelin proteins, Prefrontal cortex, Depression

## Abstract

**Background:**

The primary protein components of white matter include myelin basic protein (MBP) and 2’,3’-cyclic nucleotide 3’-phosphodiesterase (CNP). Alterations in their expression are significantly implicated in depression. This study investigated changes in MBP and CNP expression associated with depressive-like behaviors induced by chronic unpredictable stress (CUS) and evaluated therapeutic interventions using fluoxetine (FLU), an enriched environment (EE), or their combination.

**Methods:**

Male Sprague Dawley rats were randomly assigned to a control group and four CUS-exposed groups undergoing 6 weeks of stress. During the final 3 weeks of CUS, rats received daily fluoxetine (CUS + FLU group), were housed in EE (CUS + EE group), or received combined EE and fluoxetine (CUS + FLU + EE group). Depression-like behaviors were assessed through sucrose preference, forced swimming, and open field tests after CUS completion and at the end of weeks 4–6. Protein and mRNA expression levels of MBP and CNP in the prefrontal cortex were quantified via immunohistochemistry, western blot, and qRT-PCR.

**Results:**

Three weeks following CUS exposure, rats demonstrated significant depression-like behavioral phenotypes. By the fifth week, these behavioral deficits were ameliorated in the CUS + FLU + EE, whereas the CUS + FLU and CUS + EE groups exhibited comparable behavioral recovery by week 6. Parallel molecular analyses revealed diminished protein and mRNA expression levels of MBP and CNP in the prefrontal cortex of CUS-exposed animals, accompanied by a pronounced elevation in IL-1β expression. Therapeutic interventions with FLU, EE, or their combination significantly attenuated these CUS-induced molecular alterations.

**Conclusions:**

The antidepressant effects correlated with restored MBP, CNP, and IL-1β expression levels, suggesting that MBP/CNP deficiencies in depression may involve IL-1β elevation. In particular, combined enriched environment and fluoxetine accelerated behavioral recovery.

**Supplementary Information:**

The online version contains supplementary material available at 10.1186/s12993-025-00282-1.

## Introduction

Despite extensive research, the pathophysiological mechanisms underlying major depressive disorder (MDD) remain poorly understood, hampering the development of targeted therapeutic strategies. Emerging neuroimaging evidence implicates cerebral white matter abnormalities in depression pathogenesis, with diffusion tensor imaging (DTI) studies consistently demonstrating reduced white matter integrity in the prefrontal cortex (PFC) and hippocampus of both MDD patients [1–3], and chronic unpredictable stress (CUS) rodent models [4]. These findings suggest structural and functional deficits in myelination—a critical determinant of neuronal connectivity mediated by oligodendrocyte-derived myelin sheaths.

Myelin basic protein (MBP), the second most abundant component of central myelin, plays an essential role in maintaining the structural stability of myelinated axons. Clinical and preclinical studies have identified significant MBP reductions in the PFC of depressed patients [5]and chronic stress-exposed rodents [6], with evidence suggesting these deficits contribute to MDD pathophysiology. In particular, the antidepressant desvenlafaxine ameliorates both depressive behaviors and MBP loss in murine models [7], highlighting myelination restoration as a potential therapeutic mechanism. Parallel findings involve 2’,3’-cyclic nucleotide 3’-phosphodiesterase (CNP), a cytoskeletal regulator critical for oligodendrocyte maturation. Reduced CNP expression in the ventromedial PFC of MDD patients further underscores oligodendrocyte dysfunction as a hallmark of depressive neuropathology [8].

Contemporary antidepressant therapies including selective serotonin reuptake inhibitors (SSRIs), physical interventions, and behavioral approaches that exhibit variable efficacy, prompting investigation into combinatorial strategies. Enriched Environment (EE), defined as enhanced sensory-motor stimulation through novel objects and social interaction [9], demonstrates synergistic effects with SSRIs in rodent models [10]. EE confers neuroplastic benefits, including stress resilience [11] and cognitive enhancement [12], though its molecular mechanisms remain incompletely characterized.

The PFC, a neural hub governing emotional regulation and executive function, represents a critical locus for depressive pathophysiology. While SSRIs such as fluoxetine primarily address serotonergic deficits [13], emerging evidence suggests EE may enhance therapeutic outcomes through myelination-related mechanisms. However, significant knowledge gaps remain: no systematic investigations have examined whether EE potentiates fluoxetine’s ability to mitigate chronic unpredictable stress (CUS)-induced myelin protein deficits in the PFC. To address these gaps, this study had two primary objectives: To evaluate the individual and combined effects of EE and fluoxetine on CUS-induced myelin protein loss in the PFC. To investigate whether EE facilitates the accelerated onset of therapeutic efficacy in fluoxetine administration. This study integrates molecular and behavioural analyses to offer novel insights into combinatorial therapeutic strategies for myelin dysfunction. Such a mechanistic approach holds the potential to address the limitations of current monotherapies in depression.

## Materials and methods

### Animals and experimental design

**Fig. 1:**
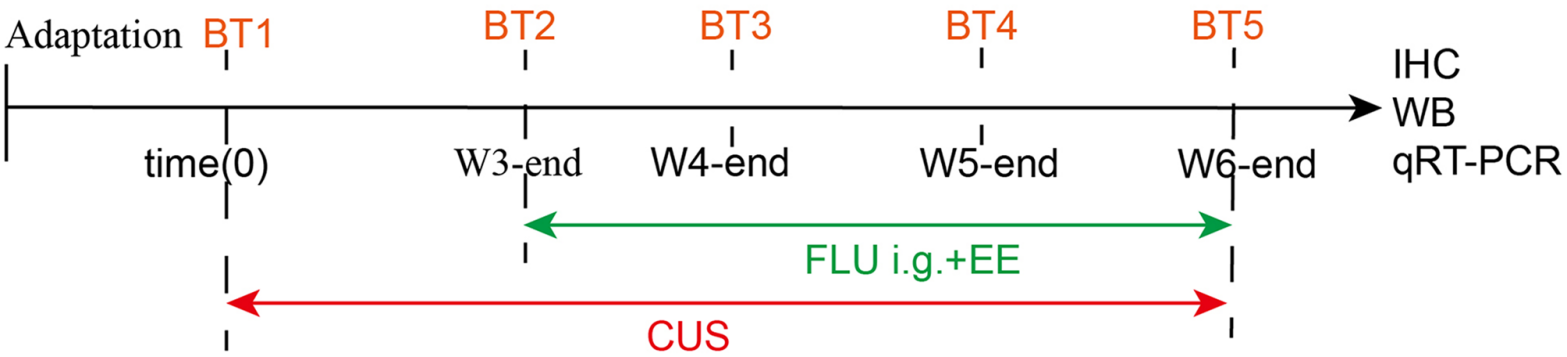
Schematic flowchart of the experimental design. BT: Behavioral tests, including the Sucrose Preference Test, Open Field Test, and Forced Swim Test. W3-end: at the end of week 3. IHC: immunohistochemistry; WB: Western-blot; qRT-PCR: Quantitative Real-Time PCR. FLU i.g.+EE: intragastric administration of fluoxetine + Enriched environment. CUS: Chronic Unpredictable Stress

Seventy male Sprague-Dawley rats (180–200 g; Experimental Animal Center of Zhengzhou University, license SCXK[Yu]2010-0002) were individually housed under controlled conditions (20–24 °C, 40–60% humidity, 12/12-hour light/dark cycle) with ad libitum access to food and water. The animals were maintained on a 12:12-hour light/dark cycle (lights on at 07:00, lights off at 19:00). All behavioural testing and treatment administrations were conducted during the light phase (08:00–16:00) to minimise circadian variability. Following assessment of spontaneous locomotor distance during standardised 5-minute open field trials, outlier identification was systematically conducted using an empirically established Z-score approach. Individual data points demonstrating absolute Z-score values exceeding the conventional threshold of 2 standard deviations (|Z| > 2) were objectively designated as statistical outliers and consequently excluded from subsequent analyses. The remaining sixty rats were randomly allocated into five experimental groups (*n* = 12/group): (1)Control: Standard housing with daily handling; (2)CUS: Chronic unpredictable stress (CUS) for 6 weeks; (3)CUS + FLU: CUS + fluoxetine (10 mg/kg/day, oral gavage, weeks 4–6); (4)CUS + EE: CUS + enriched environment (12 h/day, weeks 4–6); (5)CUS + FLU + EE: CUS + combined fluoxetine and enriched environment (weeks 4–6). The simplified process is shown in Fig. 1. All procedures were performed according to National Institute of Health (NIH) guidelines and approved by the Animal Ethics Committee of the Second Affiliated Hospital of Xinxiang Medical University(Ethics number 2015-03-18).

### Drug administration

The selected fluoxetine dose (10 mg/kg/day)aligns with established protocols for chronic antidepressant efficacy in rodent models, as referenced in prior studies [14]. According to the manufacturer’s pharmacological specifications for fluoxetine hydrochloride, the maximum aqueous solubility is formally documented at 14 mg/mL. We have clarified that fluoxetine hydrochloride (Changzhou Siyao Pharmaceuticals Co., Ltd., batch 2013–11011) was dissolved in saline (0.9% NaCl) at a concentration of 1 mg/ml. This concentration level ensured complete dissolution without precipitation, and it was freshly prepared every 48 h to ensure stability. The average daily intake was adjusted based on weekly body weight measurements. Solubility was confirmed via pilot experiments (no precipitation was observed at this concentration). The volume of fluoxetine solution was calculated based on body weight and drug concentration. The rats in the control, CUS, and CUS + EE groups received equivalent saline volumes.

### Chronic unpredictable stress (CUS) protocol

CUS-exposed rats underwent daily randomized stressors for 6 weeks, including:

(1) Foot shocks (1 mA, 10 s × 20); (2) Cold water immersion (4 °C, 5 min); (3)Cage shaking (1 Hz, 10 min); (4)Tail clamping (1 min); (5)Heat exposure (45 °C, 5 min); (6)Light/dark cycle reversal (24 h); (7)Water/food deprivation (24 h); (8)Restraint stress (2 h); (9)Wet bedding (24 h).

### Enriched environment (EE)

The EE protocol was implemented following established methods [15]. The enrichment apparatus comprised two interconnected chambers (70 cm × 60 cm × 50 cm) linked by a flexible tunnel, equipped with modular enrichment components: wooden climbing structures, running wheels, chewable rewards, manipulable objects (plastic balls, suspended swings), shelters, PVC tunnels, and abrasive whetstones. Environmental novelty was maintained by replacing damaged or soiled items twice weekly. Rats were permitted unrestricted exploration of the EE system for 12 h daily (20:00–08:00) with ad libitum access to food and water to prevent resource competition.

### Behavioral assessments

Depression-like phenotypes were evaluated using the forced swim test (FST), open field test (OFT), and sucrose preference test (SPT). Behavioral testing was conducted at baseline (pre-CUS) and weekly from weeks 3 to 6 post-intervention.

### Sucrose preference test (SPT)

Anhedonia, a core depressive symptom, was quantified via SPT as described [16]. During the initial acclimatization phase, subjects were individually housed with concurrent access to 1% sucrose solution and purified water in separate receptacles for 48 h. To mitigate positional bias, fluid container orientation was systematically altered 24 h post-acclimatization commencement. The subsequent testing phase commenced with a 4-hour hydrational deprivation period following the completion of accommodation training. Behavioural evaluation was then conducted over a 60-minute interval utilising a counterbalanced presentation paradigm, with solution containers being repositioned at 30-minute intervals to control for lateral preference artefacts. Liquid consumption was measured gravimetrically, and sucrose preference (%) was calculated as: Sucrose Preference = Sucrose Intake/ (Sucrose Intake + Water Intake)×100.

### Open field test (OFT)

The OFT was used to assess locomotor activity and exploratory behavior [17]. that analyzed in a 100 cm × 100 cm × 40 cm arena using the Smart 3.0 tracking system (Panlab Harvard Apparatus, USA). After a 30-second habituation period, 10-minute sessions were recorded. The arena was sanitized with 75% ethanol between trials.

### Forced swim test (FST)

Behavioral despair was assessed in a transparent acrylic cylinder (24 cm diameter × 50 cm height) filled to 35 cm with 25 °C water [18]. On the initial experimental day, each rodent underwent a 15-minute habituation period within the cylindrical apparatus before being subsequently removed, dried, and reintroduced to its housing cage. Following a 24-hour interval, a 5-minute videotaped trial was conducted to quantify immobility duration, defined operationally as the cessation of all volitional movement except respiratory motions essential for maintaining cranial buoyancy. The experimental protocol incorporated systematic water replenishment and 75% ethanol-based surface decontamination between successive trials to eliminate residual olfactory cues from preceding subjects. Throughout behavioural assessments, Two investigators remained blinded to the experimental cohorts of subjects.

### Immunohistochemistry (IHC)

Following terminal behavioral testing, rats (*n* = 6/group) underwent transcardial perfusion with ice-cold 4% paraformaldehyde (PFA). Brains were postfixed in 4% PFA for 12 h, cryoprotected in graded sucrose solutions (10%, 20%, 30%), and embedded in optimal cutting temperature (OCT) compound (Sakura Finetek, Japan). PFC Sect. (12 μm) were cut using a cryostat (Leica, Germany) and immunostained with: Rabbit anti-MBP (1:200, BA0094, Boster Biological, China); Rabbit anti-CNPase (1:100, bs-1000R, Bioss Antibodies, China) using a SABC kit (Boster Biological, China) per manufacturer guidelines. Four PFC sections per animal were imaged using bright field microscopy. Immunohistochemical image analysis was focused exclusively on the medial PFC subregion, which is a key neural substrate for emotion regulation in depressive pathology. The coordinates relative to the bregma and cortical surface are as follows: Anterior-Posterior (AP): +3.2 mm and + 2.7 mm; Mediolateral (ML): ±0.5 mm and ± 0.8 mm (bilateral targeting); Dorsoventral (DV): -3.2 mm to -4.5 mm (ventral to the cortical surface). Quantitative analysis was performed using Image-Pro Plus 6.0 software (200× magnification) to calculate normalised optical density values, defined as integrated optical density (IOD) divided by the region of interest area (IOD/AREA).

### Western blot (WB)

Six left PFC samples per group underwent western blot analysis. Tissue homogenisation was performed in RIPA lysis buffer followed by ice incubation (30 min) and centrifugation (12,000 × g, 15 min, 4 °C). Protein concentrations were quantified using a BCA assay (P0012, Beyotime Biotechnology, China). Samples were subsequently denatured at 95 °C for 5 min after the addition of protein sample loading buffer (LT101, Epizyme Biotech, China) and PBS buffer in proportion to protein concentrations. Electrophoresis was conducted on 12% SDS-PAGE gels under sequential voltage conditions (80 V, 30 min; 110 V, 70 min), followed by protein transfer to PVDF membranes (0.45 μm, 250 mA, 90 min).

Membranes were blocked with 5% BSA before overnight incubation at 4 °C with primary antibodies: Anti-MBP (1:1000, 10458-1-AP, Proteintech, China); Anti-CNP (1:1000, 13427-1-AP, Proteintech, China); Anti-IL-1β (1:1000, 16806-1-AP, Proteintech, China); Anti-GAPDH (1:5000, HRP-60004, Proteintech, China); Anti-α-Tubulin (1:5000, 66031-1, Proteintech, China); Secondary antibody incubation was performed for 1 h using either: HRP-conjugated Goat Anti-Rabbit IgG(H + L) (1:1000, SA00001-2, Proteintech, China); HRP-conjugated Goat Anti-Mouse IgG(H + L) (1:1000, SA00001-1, Proteintech, China). Following development with the high-sensitivity ECL substrate (Cytiva, UK), western blot images were captured using an Amersham Imager 600 system (Cytiva) with integrated band intensity quantification.

### Quantitative real-time PCR (qRT-PCR)

For qRT-PCR analysis, six right PFC samples per group were processed. PFC tissues were dissected on dry ice, snap-frozen in liquid nitrogen, and stored at -80 °C. Total RNA was isolated using TRIzol (15596026, Invitrogen, USA), quantified via spectrophotometry (NanoDrop 2000, Thermo Fisher, USA), and reverse-transcribed (1 µg RNA) using a cDNA synthesis kit. qRT-PCR was performed on a QuantStudio 6 Flex system (Applied Biosystems, USA) with 20 µL reactions containing: 2 µL cDNA; 0.4 µM forward/reverse primers (Table 1);10 µL SYBR Green Master Mix (U8305, Tiangen Biotech, China);6 µL nuclease-free water Cycling parameters: 95 °C (30 s); 40 cycles of 95 °C (5 s), 60 °C (30 s), 72 °C (30 s). GAPDH expression was employed as the endogenous reference for normalisation. Relative mRNA expression levels of target genes (MBP, CNP, IL-1β) were calculated using the comparative 2 − ΔΔCt method.

**Table 1:** The sequence of primers

Primers	Sequence(Forward)	Sequence(Reverse)
GAPDH	5′-ATGGCTACAGCAACAGGGT − 3’	5′-TTATGGGGTCTGGGATGG-3’
MBP	5′-ACCACTCTGGAAAGCGAGAATTAGC-3’	5′-ACTGTCTTCTGAGGCGGTCTGAG-3’
CNP	5′-AAGTACCACAACGGCACCAAGATG-3’	5′-CGAGCACAAGAACCCTGATGTCC-3’
IL−1β	5’-CCTTGTGCAAGTGTCTGAAGC-3’	5’-CCCAAGTCAAGGGCTTGGAA-3’

### Statistical analysis

All datasets underwent rigorous normality assessment using Shapiro-Wilk tests (α = 0.05) and homogeneity of variance verification through Levene’s test before parametric analysis. Behavioural parameters measured across baseline and post-treatment intervals were analysed via repeated-measures ANOVA with Mauchly’s Test of Sphericity and Bonferroni-corrected post hoc comparisons, while between-group differences in MBP, CNP, and IL-1β expression levels were evaluated through one-way ANOVA. Continuous variables are expressed as mean ± standard deviation (SD). The threshold for statistical significance was maintained at two-tailed *P* < 0.05 throughout all analyses. All statistical procedures were implemented using GraphPad Prism (9.0, USA).

## Results

### Combined FLU and EE intervention reverses depression-like behaviors in CUS-induced rats

**Fig. 2:**
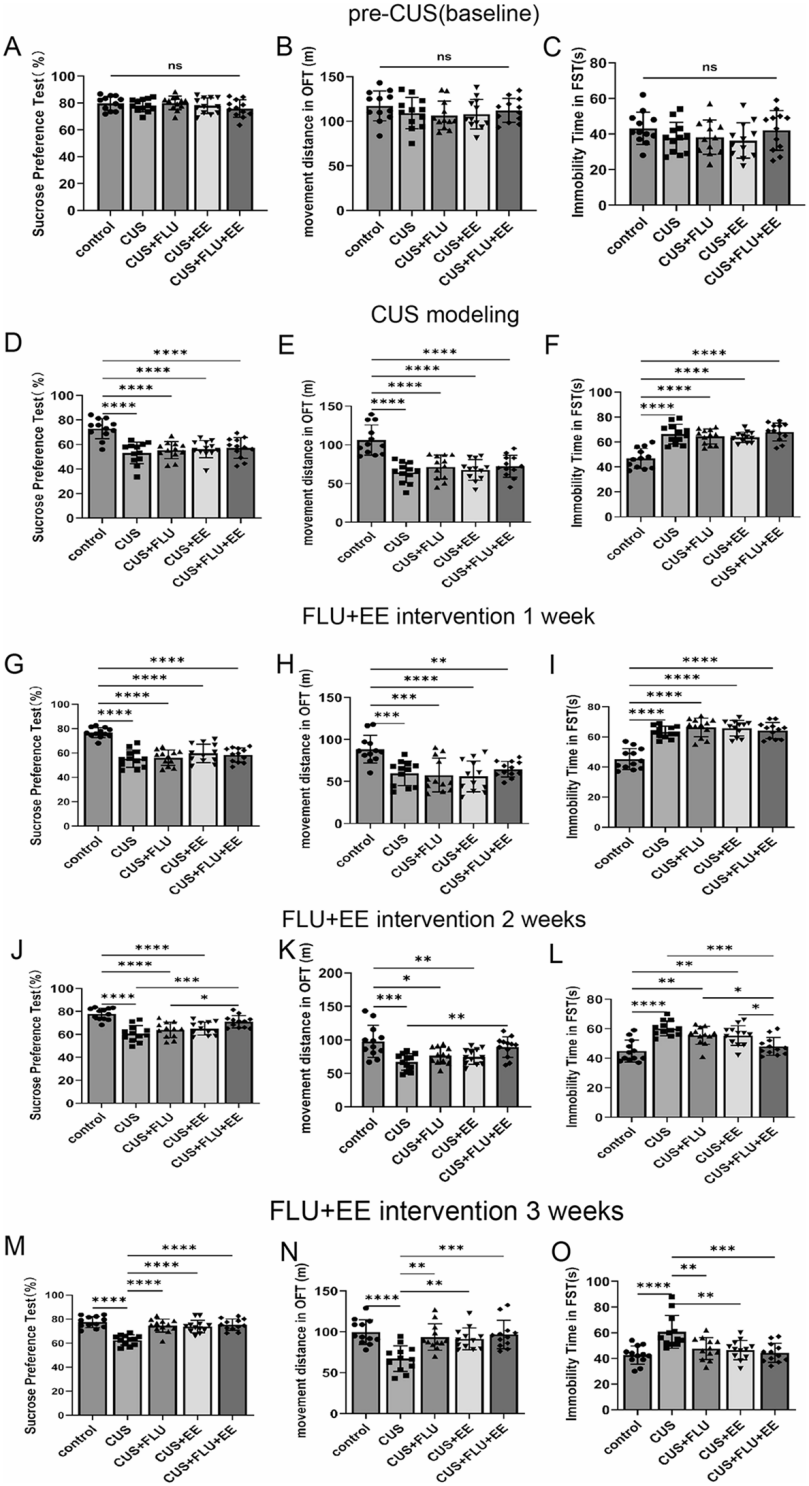
Combined FLU and EE intervention reverses depression-like behaviors in CUS-induced rats. **A**-**C**. Behavioral assessment at baseline. (**A**) sucrose preference ratio; (**B**) Movement distance in the open field. (**C**) Immobility time in forced swimming test. **D**-**F**. Behavioral assessment after CUS modeling. **G**-**I**. Behavioral assessment 1 week after treatment with fluoxetine and enriched environment. **J**-**L**. Behavioral assessment 2 weeks after treatment with fluoxetine and enriched environment. **M**-**O**. Behavioral assessment 3 weeks after treatment with fluoxetine and enriched environment. Significant differences were revealed with ^*^P < 0.05, ^**^P < 0.01, ^***^P < 0.001, ^****^P < 0.0001, *n* = 12

Repeated-measures ANOVA demonstrated that all behavioural endpoints (sucrose preference, locomotor activity in the open field test, and immobility time during the forced swim test) satisfied the assumptions of Mauchly’s Test of Sphericity (*p* > 0.05). The baseline period results revealed no statistically significant differences in behavioural measures between the experimental groups (*p* > 0.05; Fig. 2A–C). Following 3 weeks of CUS exposure, significant intergroup differences were observed in sucrose preference rate [*F*(4,55) = 12.22, *p* < 0.01], movement distance [*F*(4,55) = 14.87, *p* < 0.01], and immobility time [*F*(4,55) = 19.41, *p* < 0.01]. However, no behavioral distinctions were detected among the four CUS-exposed subgroups (*p* > 0.05). All CUS-exposed groups exhibited marked behavioral deviations from the control group (*p* < 0.05; Fig. 2D–F), confirming the successful induction of depression-like phenotypes.

By week 4 of CUS, sustained group differences persisted in sucrose preference [*F*(4,55) = 23.49, *p* < 0.01], movement distance [*F*(4,55) = 8.08, *p* < 0.01], and immobility time [*F*(4,55) = 30.27, *p* < 0.01], with continued homogeneity across CUS subgroups (*p* > 0.05; Fig. 2G–I).

At week 5, significant intergroup variance remained for sucrose preference [*F*(4,55) = 15.34, *p* < 0.01], movement distance [*F*(4,55) = 7.36, *p* < 0.01], and immobility time [*F*(4,55) = 11.77, *p* < 0.01]. The CUS + FLU + EE cohort demonstrated substantial behavioral recovery relative to untreated CUS rats (*p* < 0.01), Moreover, no statistically significant differences were observed compared to the control group (*p* > 0.05). Rats in the CUS + FLU + EE group exhibited significantly reduced immobility time in the forced swim test compared to both the CUS + FLU and CUS + EE groups (*p* < 0.05). Furthermore, sucrose preference was significantly elevated in the CUS + FLU + EE group relative to the CUS + FLU group (*p* < 0.05). These results indicate that the depressive-like phenotypes in the CUS + FLU + EE rats were effectively normalised (Fig. 2J–L).

By week 6, all intervention groups (CUS + FLU, CUS + EE, CUS + FLU + EE) exhibited restored sucrose preference and movement distance comparable to controls (*p* > 0.05), alongside significantly reduced immobility versus CUS rats (*p* < 0.05). These data indicate complete behavioral normalization following combined FLU + EE treatment (Fig. 2M–O).

### Post-treatment immunohistochemical analysis of MBP and CNP in PFC

**Fig. 3:**
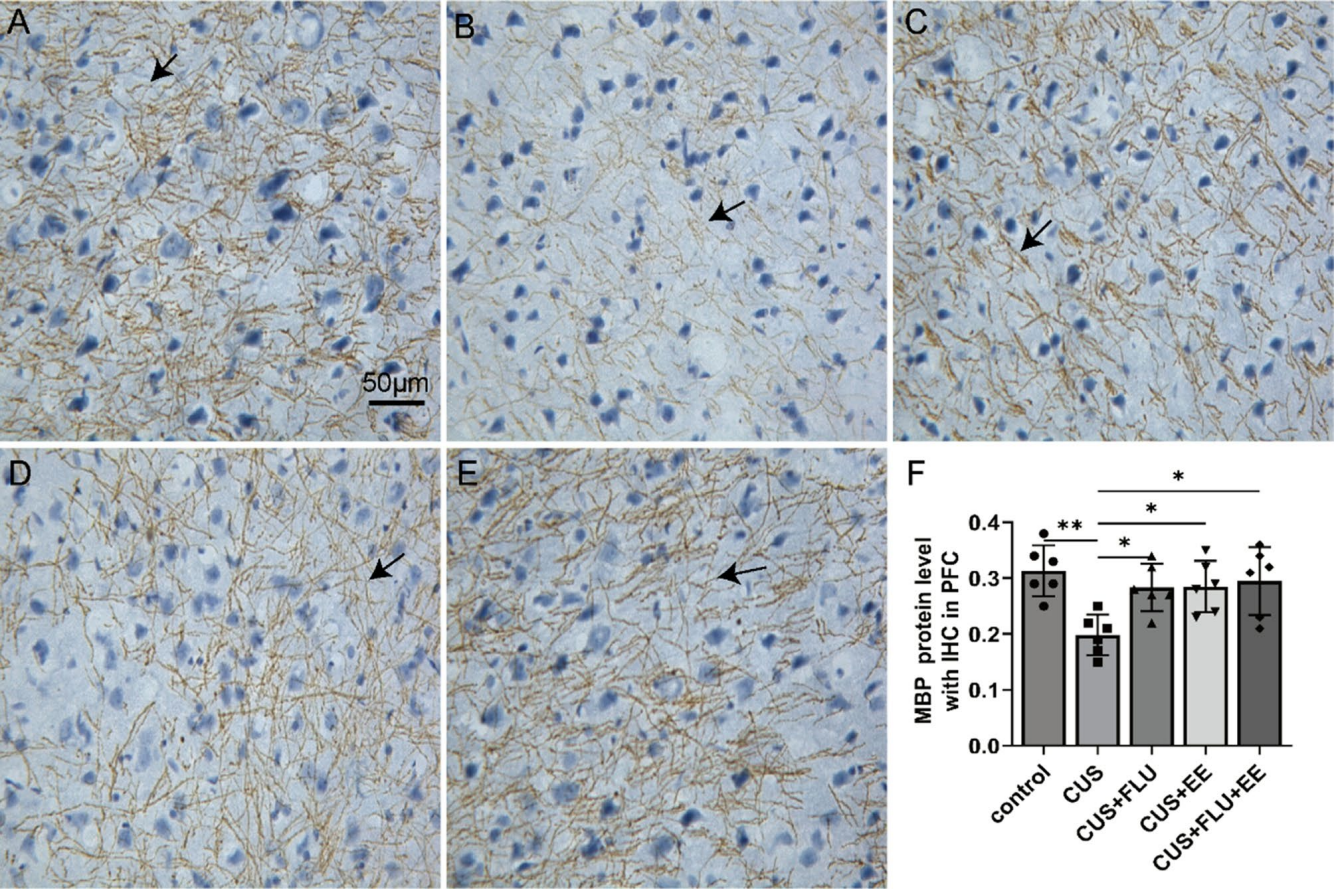
Post-treatment immunostaining intensity of MBP protein with immunohistochemistry in medial PFC. **A** MBP protein in the control group; **B**. MBP protein in the CUS group; **C**. MBP protein in the CUS + FLU group; **D**. MBP protein in the CUS + EEgroup; **E**. MBP protein in the CUS + FLU + EE group. **F**. Immunohistochemical Quantification of MBP Protein in medial PFC. All scale bars represent 50 μm; “→”: positive staining in representative medial PFC. Significant differences were revealed with ^*^P < 0.05, ^**^P < 0.01, *n* = 6

**Fig. 4:**
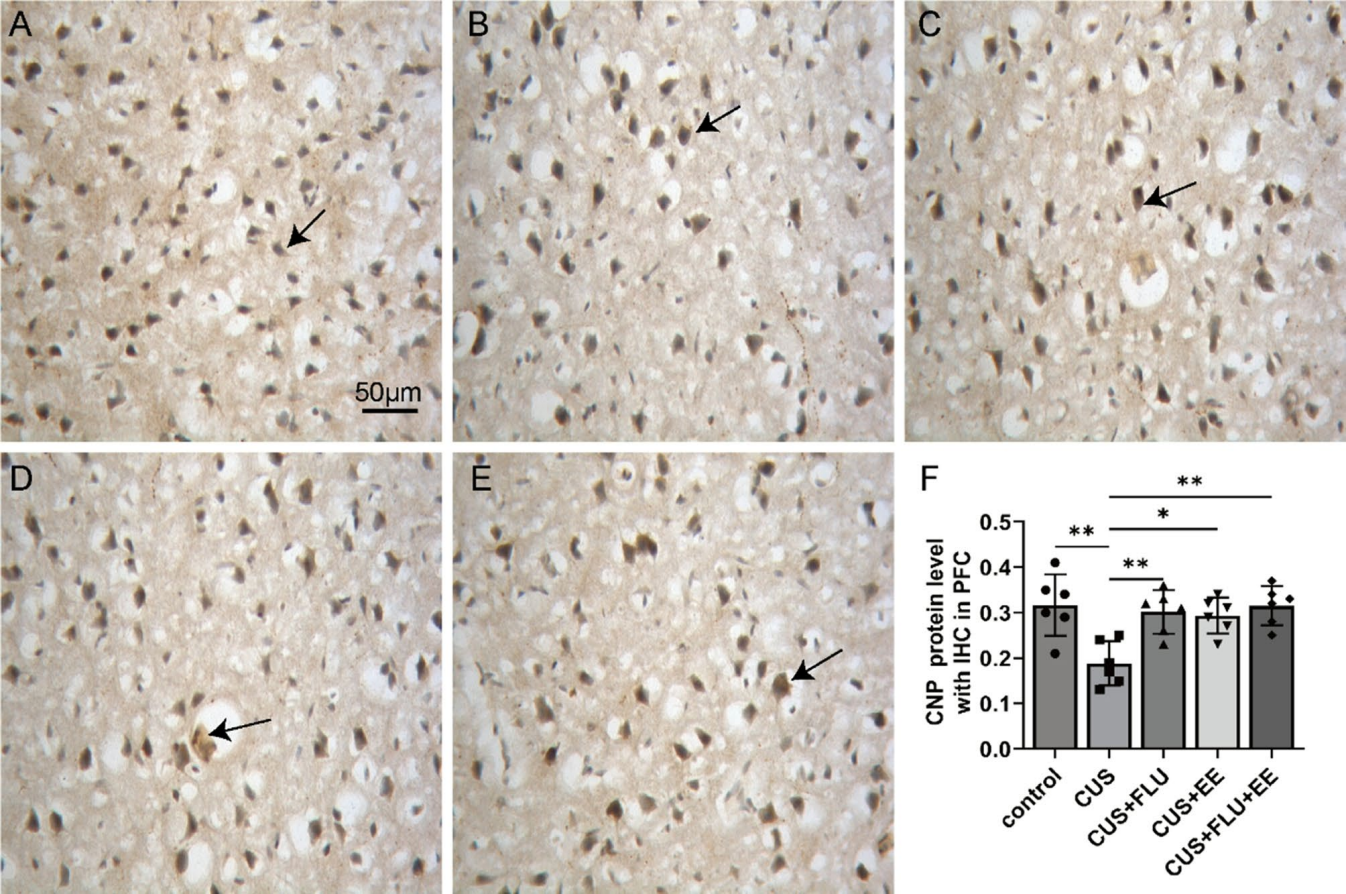
Post-treatment immunostaining intensity of CNP protein with immunohistochemistry in medial PFC. **A** CNP protein with IHC in the control group; **B**. CNP protein with IHC in the CUS group; **C**. CNP protein with IHC in the CUS + FLU group; **D**. CNP protein with IHC in the CUS + EE group; **E**. CNP protein with IHC in the CUS + FLU + EE group. **F**. Immunohistochemical Quantification of CNP Protein in medial PFC. All scale bars represent 50 μm; “→”: positive staining in representative medial PFC. Significant differences were revealed with^*^P < 0.05, ^**^P < 0.01, *n* = 6

Immunohistochemical analysis demonstrated statistically significant intergroup variations in MBP [*F*(4,25) = 5.39, *p* < 0.01] and CNP [*F* (4,25) = 6.89, *p* < 0.01] immunoreactivity within the medial prefrontal cortex (mPFC). Compared with control specimens, chronic unpredictable stress (CUS)-exposed rats exhibited markedly reduced MBP and CNP expression (*p* < 0.05). All intervention cohorts (CUS + FLU, CUS + EE, and CUS + FLU + EE) demonstrated restored protein expression levels comparable to the control group (*p* > 0.05), with no statistically significant differences detected between the intervention groups themselves (*p* > 0.05; Figs. 3 and 4).

### Post-treatment Western blot analysis of MBP, CNP, and IL-1β in PFC

**Fig. 5:**
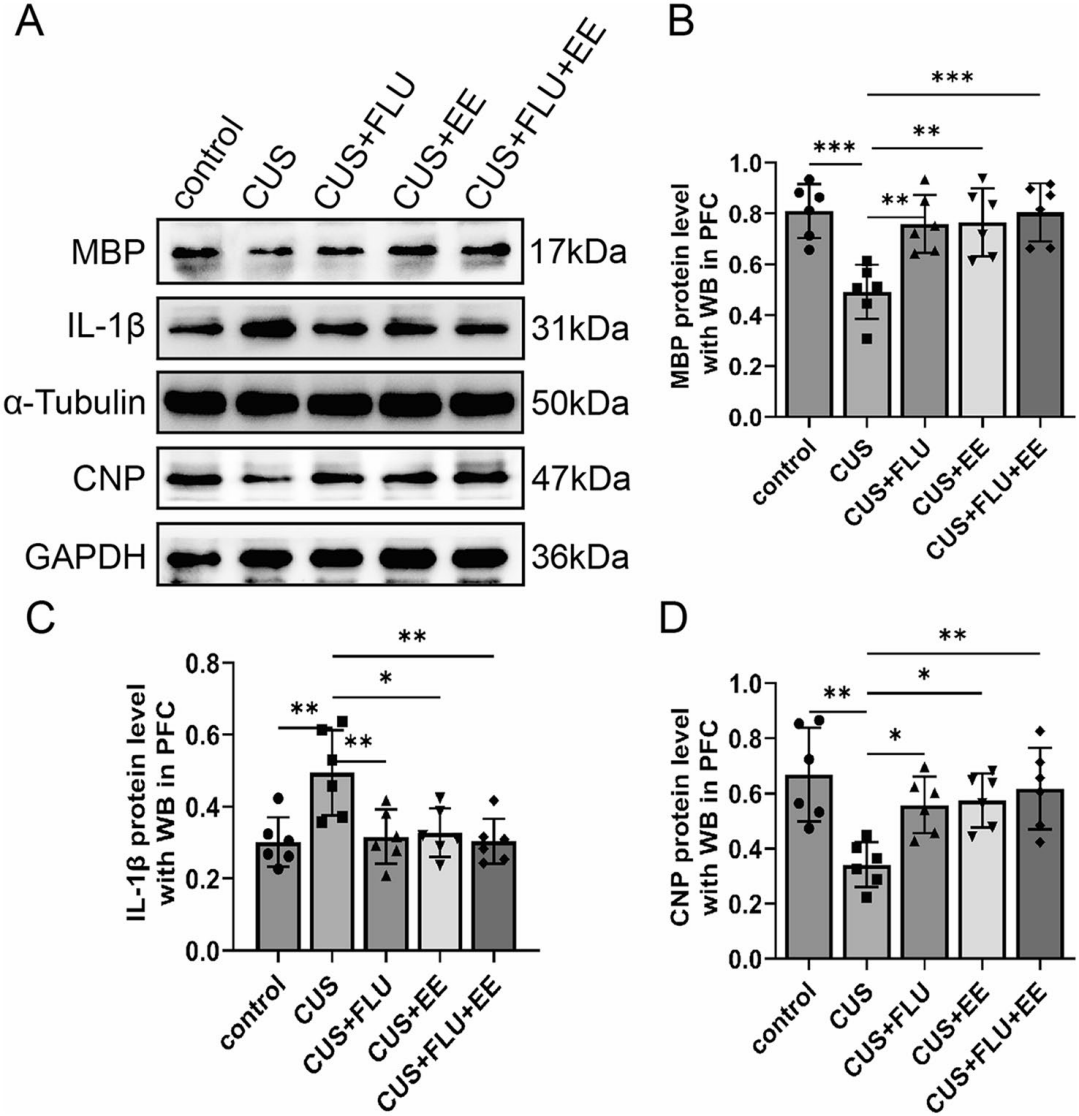
Post-treatment protein expression levels of MBP, CNP, and IL-1βwith western-blot in PFC. **A** Western blot bands of MBP, CNP, and IL-1βin PFC; **B**. Quantitative Analysis of MBP Protein by western blot; **C**. Quantitative Analysis of IL-1β Protein by western blot; **D**. Quantitative Analysis of CNP Protein by western blot. Significant differences were revealed with^*^P < 0.05, ^**^P < 0.01, ^***^P < 0.001, *n* = 6

Western blot quantification revealed significant treatment-mediated alterations in the prefrontal cortical expression of MBP [*F*(4,25) = 7.96, *p* < 0.01], CNP [*F*(4,25) = 6.07, *p* < 0.01], and IL-1β [*F*(4,25) = 6.16, *p* < 0.01]. CUS-exposed rats exhibited significantly reduced MBP/CNP expression concomitant with elevated IL-1β levels compared to control counterparts (*p* < 0.01). All therapeutic cohorts (CUS + FLU, CUS + EE, and CUS + FLU + EE) demonstrated complete normalisation of these protein expression profiles, achieving levels statistically indistinguishable from controls (*p* > 0.05) while remaining significantly differentiated from CUS specimens (*p* < 0.01). Crucially, no significant interventional disparities in MBP, CNP, or IL-1β expression were observed across the therapeutic groups (*p* > 0.05), indicating equivalent efficacy in reversing CUS-induced neurochemical disturbances (Fig. 5).

### Post-treatment mRNA expression of MBP, CNP, and IL-1β in PFC

**Fig. 6:**
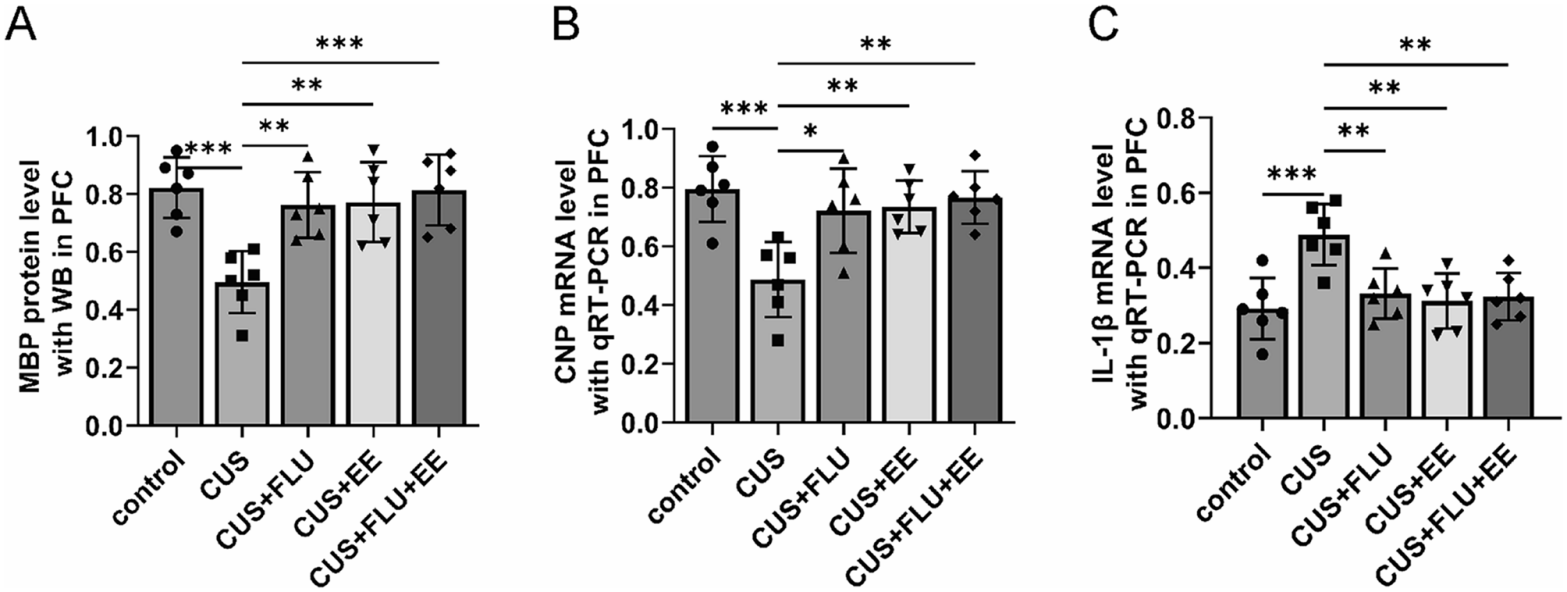
Post-treatment mRNA expression levels of MBP, CNP, and IL-1β in PFC. **A** Post-treatment mRNA level of MBP with qRT-PCR; **B**. Post-treatment mRNA level of CNP with qRT-PCR; **C**. Post-treatment mRNA level of IL-1β with qRT-PCR; Significant differences were revealed with ^*^P < 0.05, ^**^P < 0.01, ^***^P < 0.001, *n* = 6

Quantitative RT-PCR analyses revealed concordant transcriptional regulation of myelination-related markers with corresponding protein expression profiles. Therapeutic cohorts (CUS + FLU, CUS + EE, and CUS + FLU + EE) demonstrated significantly elevated MBP and CNP mRNA levels alongside reduced IL-1β transcription compared to CUS-exposed rats (*p* < 0.05 for all comparisons). mRNA expression patterns in intervention groups showed complete restoration to physiological baselines, with no statistically significant deviations from the control group (*p* > 0.05). Furthermore, no statistically significant disparities were detected in prefrontal cortical mRNA expression of MBP, CNP, or IL-1β across the therapeutic intervention groups themselves (*p* > 0.05), confirming equivalent transcriptional efficacy with EE and fluoxetine (Fig. 6).

## Discussion

Chronic stress reliably induces depression-like phenotypes in rodents, as demonstrated by prolonged immobility in forced swim tests, anhedonia (reduced sucrose preference), and hypoactivity in open field tests [19, 20]. In this study, 21 days of CUS elicited analogous behavioural deficits in rats, including despair-like immobility, diminished reward sensitivity, and motor retardation. These phenotypes closely resemble the clinical manifestations of MDD. Both EE and fluoxetine administration effectively reversed these behavioral abnormalities, with the combined intervention (EE + fluoxetine) producing the most rapid therapeutic response. This acceleration of antidepressant effects holds clinical relevance, as delayed onset of action in conventional SSRIs often compromises treatment adherence and patient outcomes [21]. The synergistic behavioral improvements observed with EE + fluoxetine align with prior evidence that environmental modulation enhances antidepressant efficacy [22]. Specifically, EE has been shown to reduce anxiety, mitigate social withdrawal, and restore exploratory behavior in stress-exposed rodents [23, 24], corroborating our findings.

At the neurobiological level, CUS-induced behavioural deficits co-occurred with PFC reductions in MBP and CNP expression, which are key myelin-associated proteins. Functional impairment of these proteins may mechanistically contribute to altered prefrontal connectivity [25]. These molecular alterations mirror postmortem observations in MDD patients, including myelin sheath abnormalities and downregulation of myelin-associated genes [4, 8, 26]. Remarkably, fluoxetine, EE, and their combination restored MBP and CNP expression in parallel with behavioral recovery, implicating myelin dysfunction in depressive pathophysiology. The superior efficacy of EE combined fluoxetine in upregulating myelin proteins suggests combinatorial neuroplasticity mechanisms, potentially involving oligodendrocyte precursor cell proliferation or differentiation [27]. Studies have demonstrated that running exercise significantly increases the expression of MBP and CNP protein in the PFC of CUS rat models [28]. The restoration of MBP and CNP protein levels observed in the CUS rats exposed to an enriched environment in the present investigation may be attributed to enhanced locomotor activity and increased exercise engagement in these rodents. Supporting this, DTI studies confirm that fluoxetine enhances white matter integrity in depressed patients [29], while EE promotes axonal repair and myelinated fiber regeneration in rodent models [30, 31]. Furthermore, the sustained antidepressant effects of ketamine appear to be mechanistically linked to its capacity to promote myelination in the mPFC of mice subjected to chronic social defeat stress [32]. These findings from these investigations provide robust evidence supporting a significant association between reduced levels of myelin-associated proteins and the pathogenesis of depression.

Mechanistic analyses indicate that both EE and the antidepressant effect of ketamine attenuated pro-inflammatory cytokine activity, particularly IL-1β, which is known to disrupt oligodendrocyte function and exacerbate demyelination [33, 34], with elevated IL-1β and TNF-α impairing myelination via enhanced monoamine reuptake and suppressed neurotrophic signalling [35, 36]. Consistent with existing data, IL-1β suppression, and related anti-inflammatory interventions restore CNP expression and myelin architecture in experimental multiple sclerosis models [37]. Critically, however, the observed reduction in IL-1β, concurrent with MBP/CNP alterations in treated groups, constitutes correlational evidence rather than causal proof. Existing literature documents that pharmacological anti-inflammatory agents (e.g., minocycline) and CX3CR1 pathway modulation alleviate depressive phenotypes through microglial deactivation, IL-1β suppression, and subsequent oligodendrocyte precursor cell (OPC) maturation in striatal-hippocampal circuits [38, 39]. Our laboratory’s preceding work further established that EE mitigates depressive behaviours in CUS rats via inhibition of microglial M1-polarisation and IL-1β downregulation [40]. Building upon the contextual alignment of these independent findings with our observed synchronised suppression of IL-1β levels and restoration of myelination processes in the PFC of CUS-exposed rats, this neurochemical synchronisation coincided with behavioural recovery from depressive-like phenotypes. It is proposed that EE and fluoxetine exert neuroprotective effects through a coordinated mechanistic framework involving the modulation of pro-inflammatory signalling cascades and transcriptional regulation of myelin-associated genes. Such combinatorial effects may potentiate myelinization while enhancing synaptic transmission efficacy, though causal relationships require empirical verification through targeted pathway interrogation.

### Limitations

1. Behavioural specificity: Stress/treatment effects on swimming/climbing behaviours were not assessed, potentially compromising FST validity.
2. Longitudinal scope: While baseline homogeneity was confirmed pre-CUS, the design omitted longitudinal stress resilience tracking and formal criteria for treatment non-response.
3. Mechanistic causality: The causal role of inflammatory-mediated oligodendrocyte impairment requires validation through lineage-tracing and cytokine knockout models to confirm IL-1β–myelin restoration links and exclude compensatory mechanisms.
4. Temporal resolution: Unanalysed temporal variations in recovery and molecular responses (e.g., MBP/CNP) preclude determining if fluoxetine/EE efficacy differed between early and late intervention phases.

## Conclusions

In summary, this study demonstrates that EE potentiates fluoxetine’s antidepressant effects by accelerating behavioral recovery and normalizing PFC myelin protein expression. The reversal of MBP/CNP deficits and IL-1β reduction suggests that myelin dysregulation in depression involves inflammatory-mediated oligodendrocyte impairment. These findings highlight the therapeutic potential of combinatorial strategies targeting both neuroinflammation and white matter plasticity in MDD.

## Electronic supplementary material

Below is the link to the electronic supplementary material.

Supplementary Material 1

## Data Availability

The datasets in this study are reliable and have been included in the supplementary materials.
