# Supplementary figures and images for: Combined enriched environment and fluoxetine enhance myelin protein expression in the prefrontal cortex of a chronic unpredictable stress depression model
Source: Behav Brain Funct. 2025 Jun 11;21:16. doi: 10.1186/s12993-025-00282-1 (PMC12160429; doi:10.1186/s12993-025-00282-1)

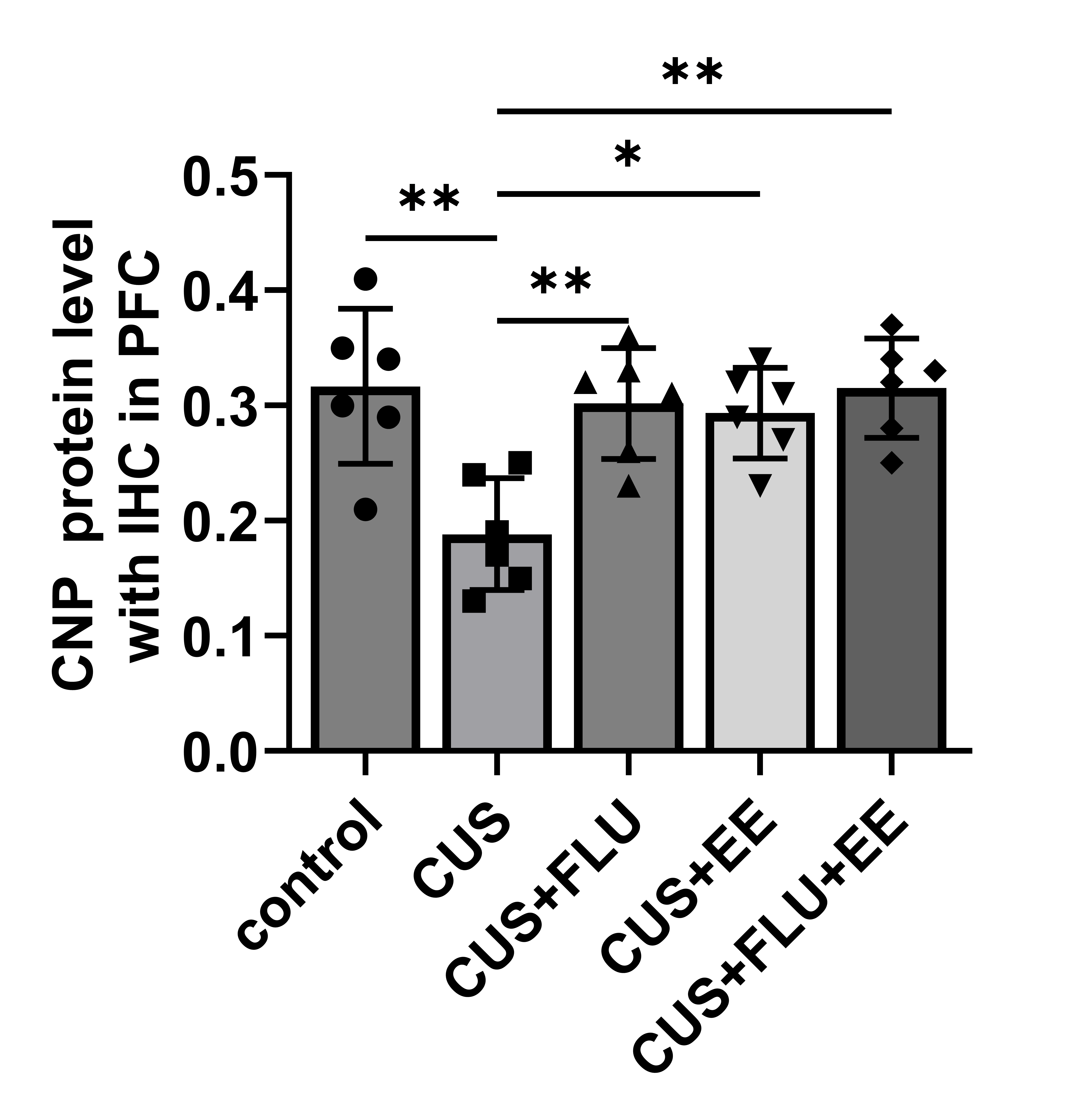

Supplement: Supplementary file 1 — Supplementary Material 1 [file 12993_2025_282_MOESM1_ESM.zip › IHC figure/CNP/CNP IHC.tif]

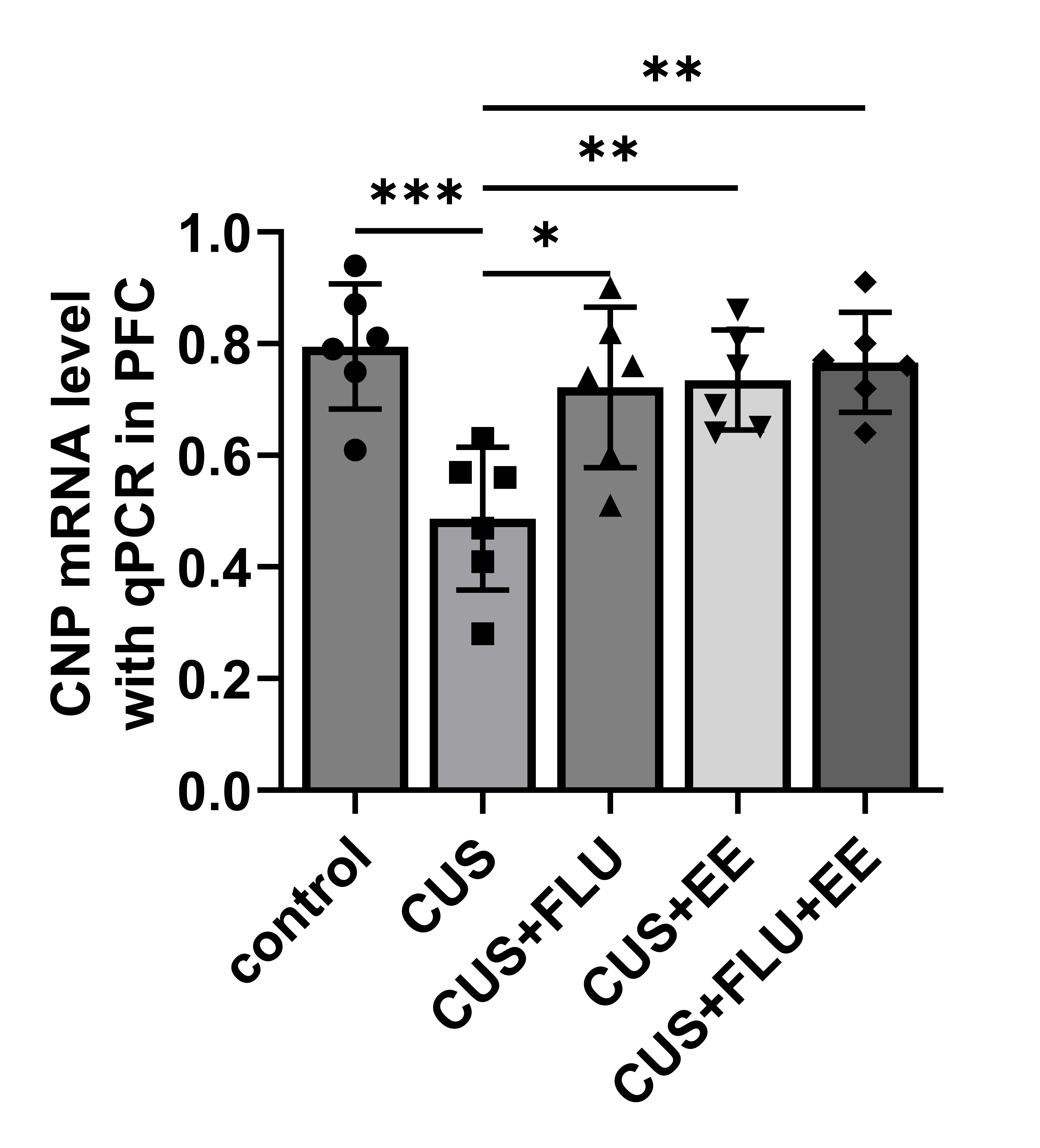

Supplement: Supplementary file 1 — Supplementary Material 1 [file 12993_2025_282_MOESM1_ESM.zip › IHC figure/CNP/CNP mRNA.tif]

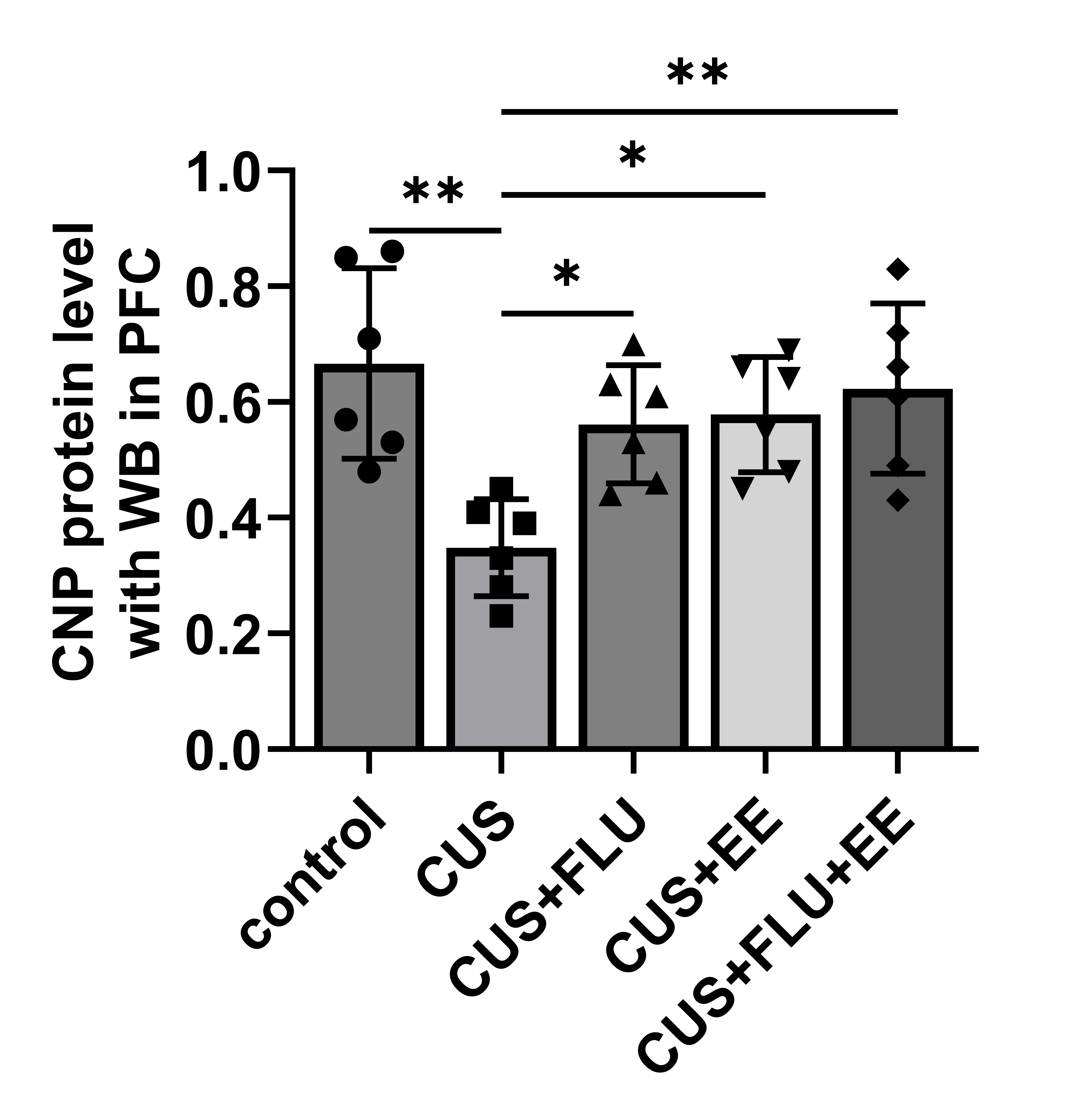

Supplement: Supplementary file 1 — Supplementary Material 1 [file 12993_2025_282_MOESM1_ESM.zip › IHC figure/CNP/CNP WB.tif]

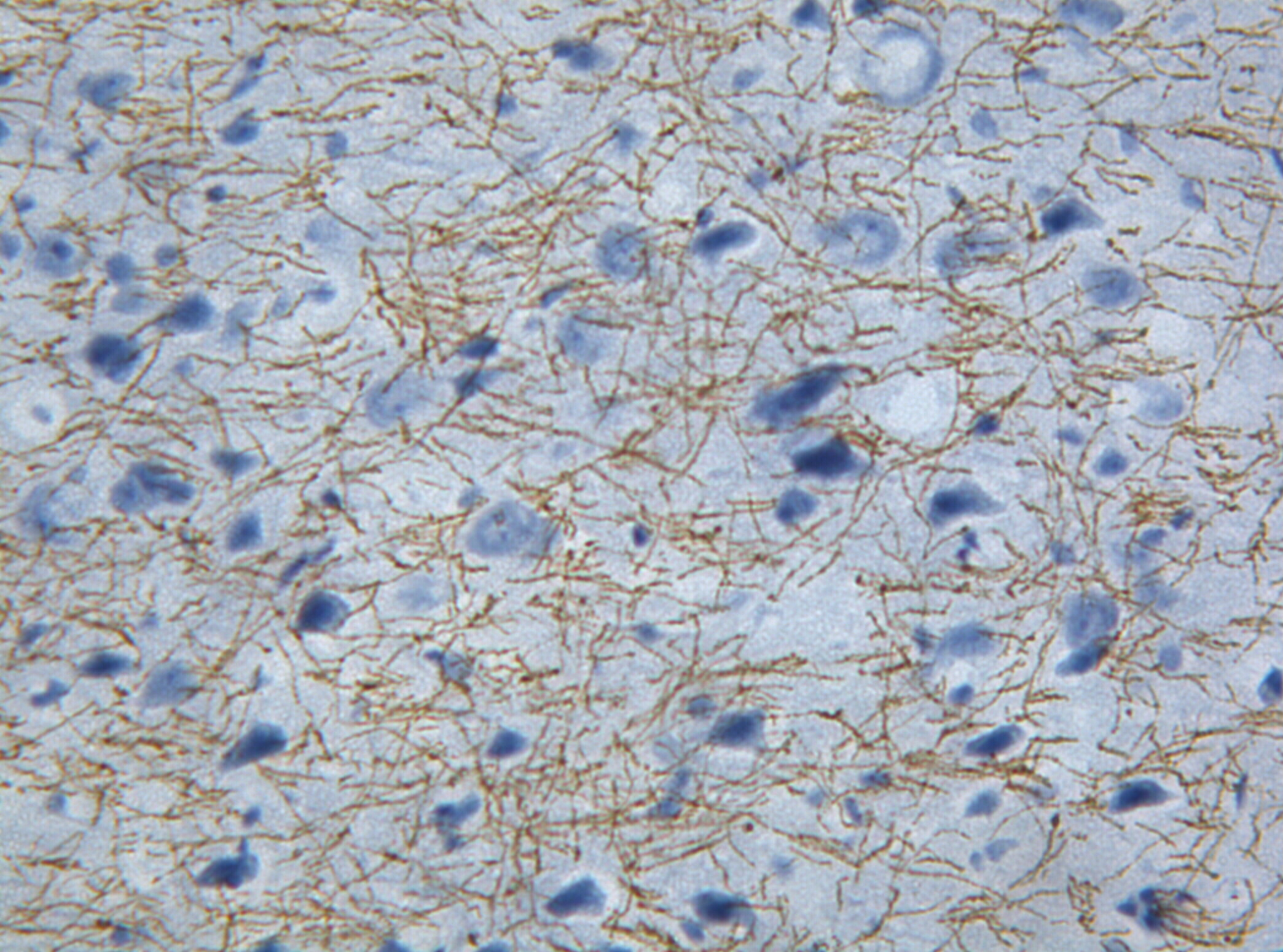

Supplement: Supplementary file 1 — Supplementary Material 1 [file 12993_2025_282_MOESM1_ESM.zip › IHC figure/MBP/control.jpg]

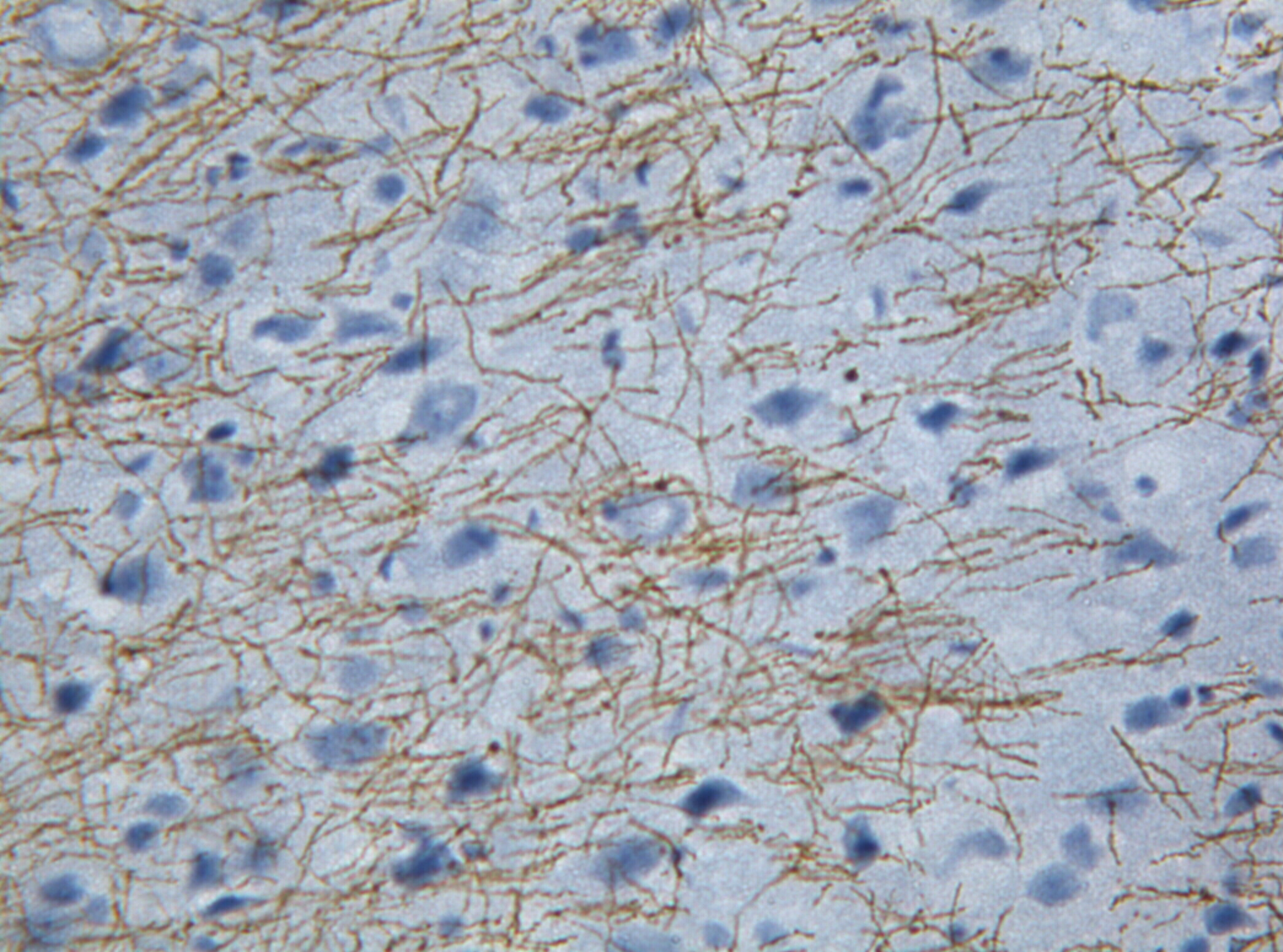

Supplement: Supplementary file 1 — Supplementary Material 1 [file 12993_2025_282_MOESM1_ESM.zip › IHC figure/MBP/CUS+EE+FLU.jpg]

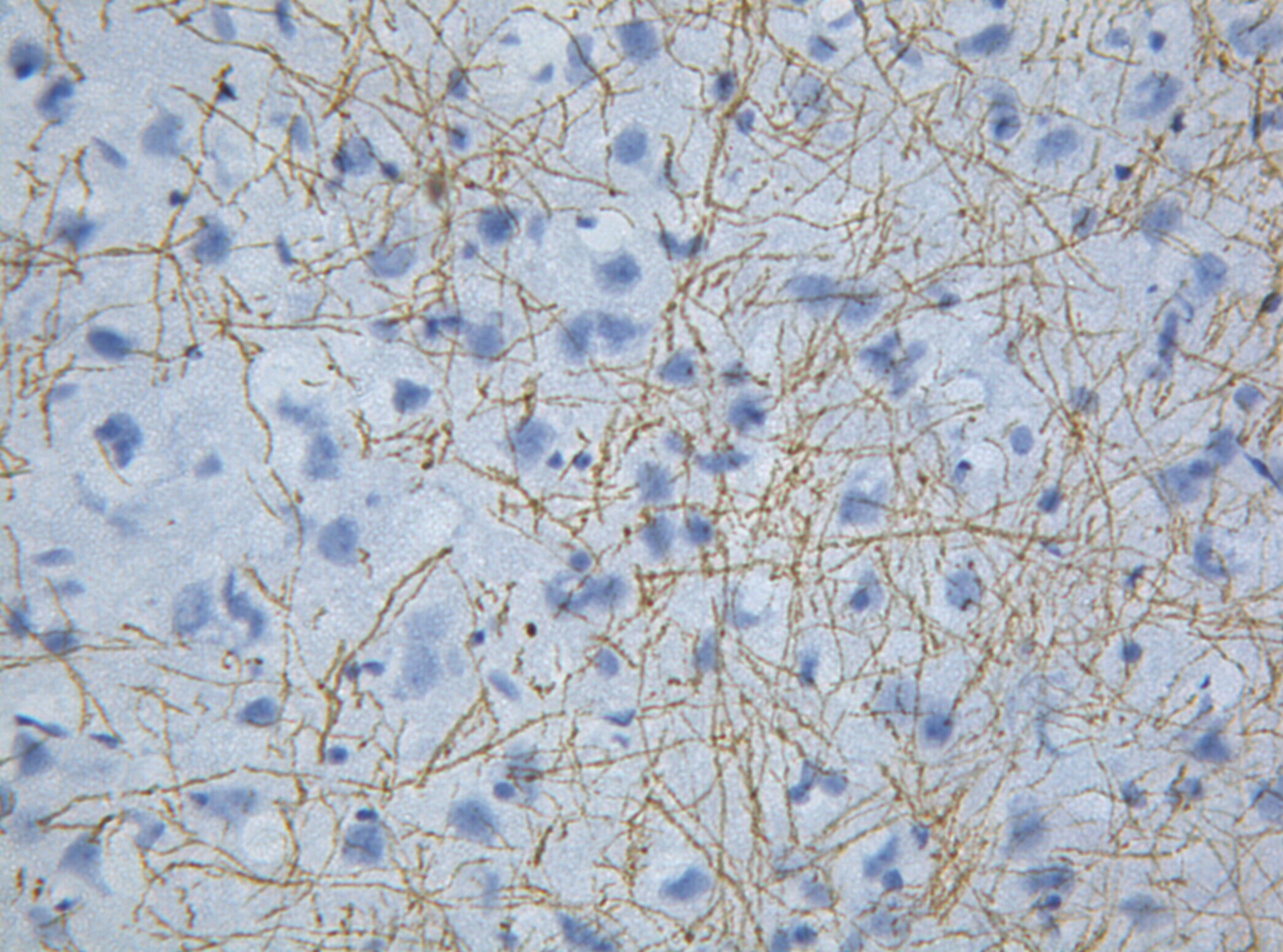

Supplement: Supplementary file 1 — Supplementary Material 1 [file 12993_2025_282_MOESM1_ESM.zip › IHC figure/MBP/CUS+EE.jpg]

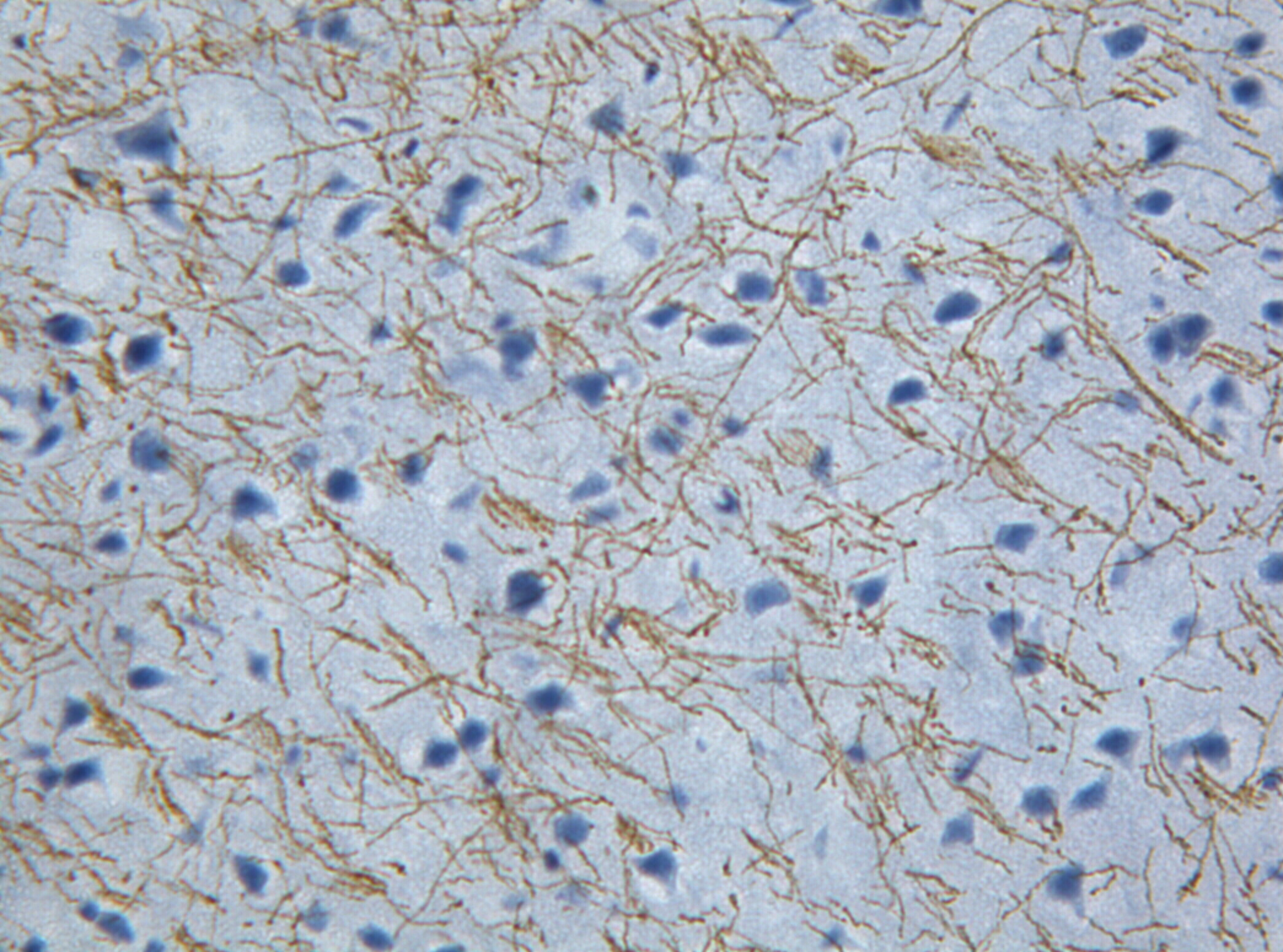

Supplement: Supplementary file 1 — Supplementary Material 1 [file 12993_2025_282_MOESM1_ESM.zip › IHC figure/MBP/CUS+FLU.jpg]

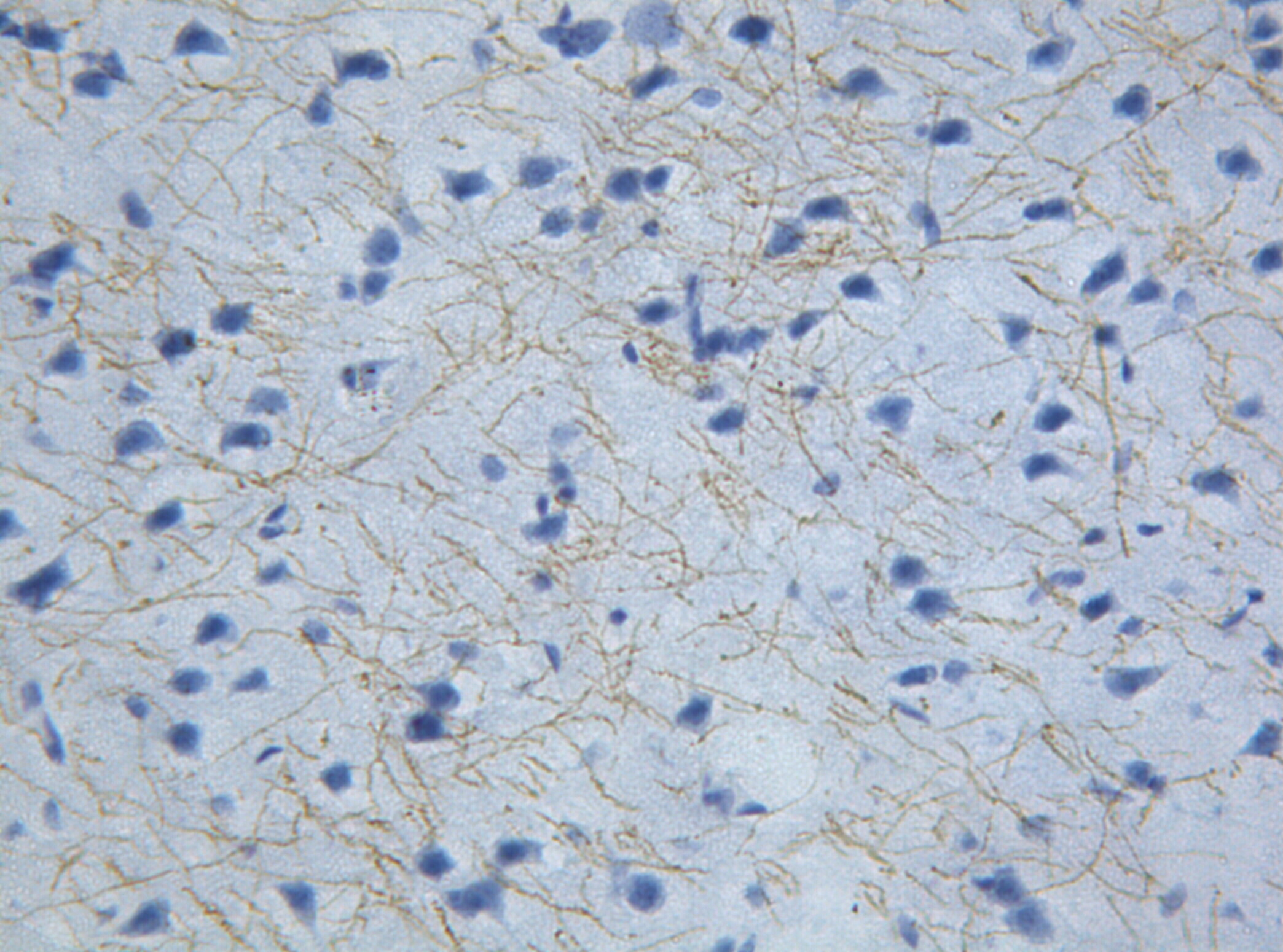

Supplement: Supplementary file 1 — Supplementary Material 1 [file 12993_2025_282_MOESM1_ESM.zip › IHC figure/MBP/CUS.jpg]

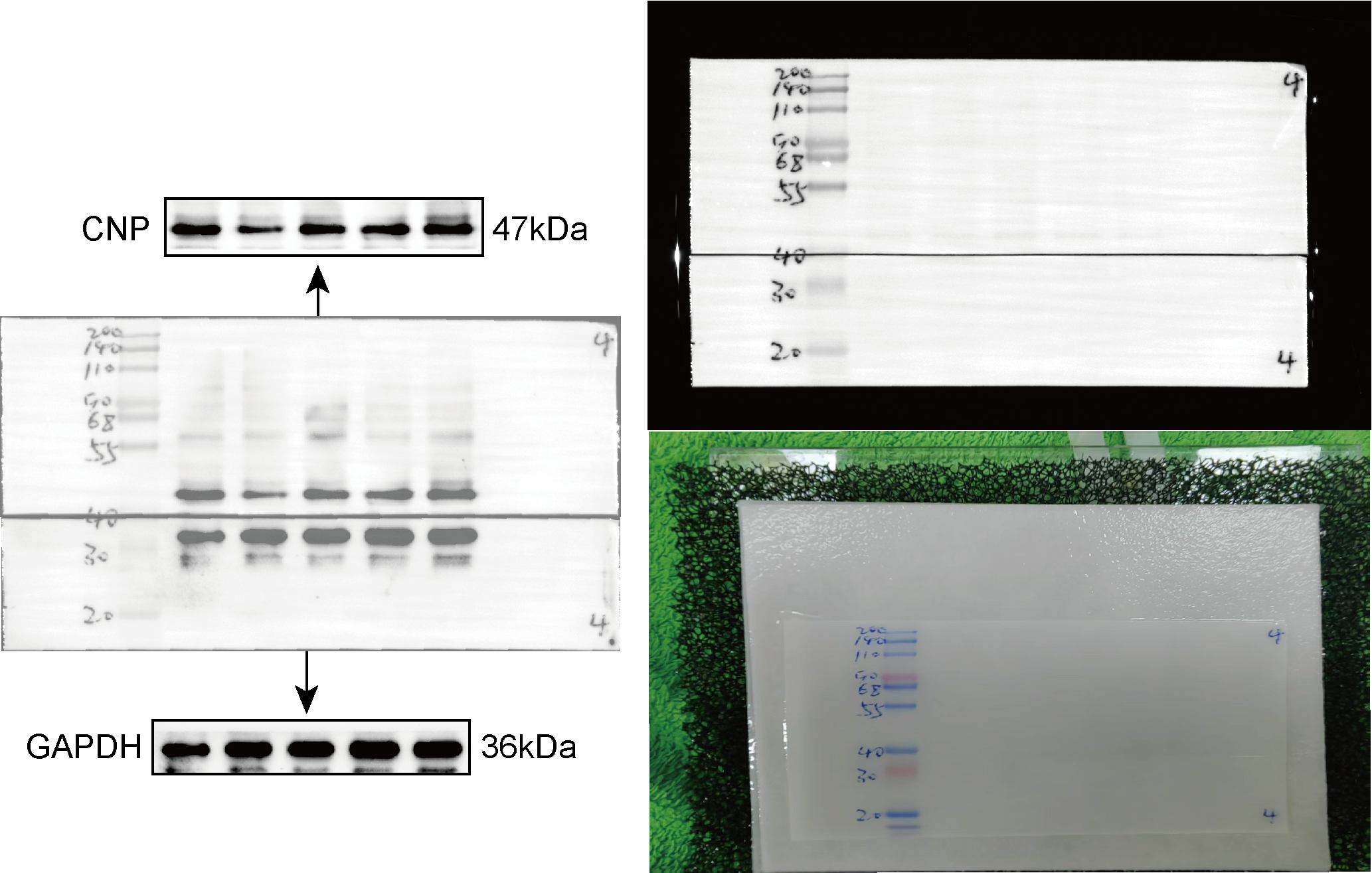

Supplement: Supplementary file 1 — Supplementary Material 1 [file 12993_2025_282_MOESM1_ESM.zip › WB figure/CNP.tif]

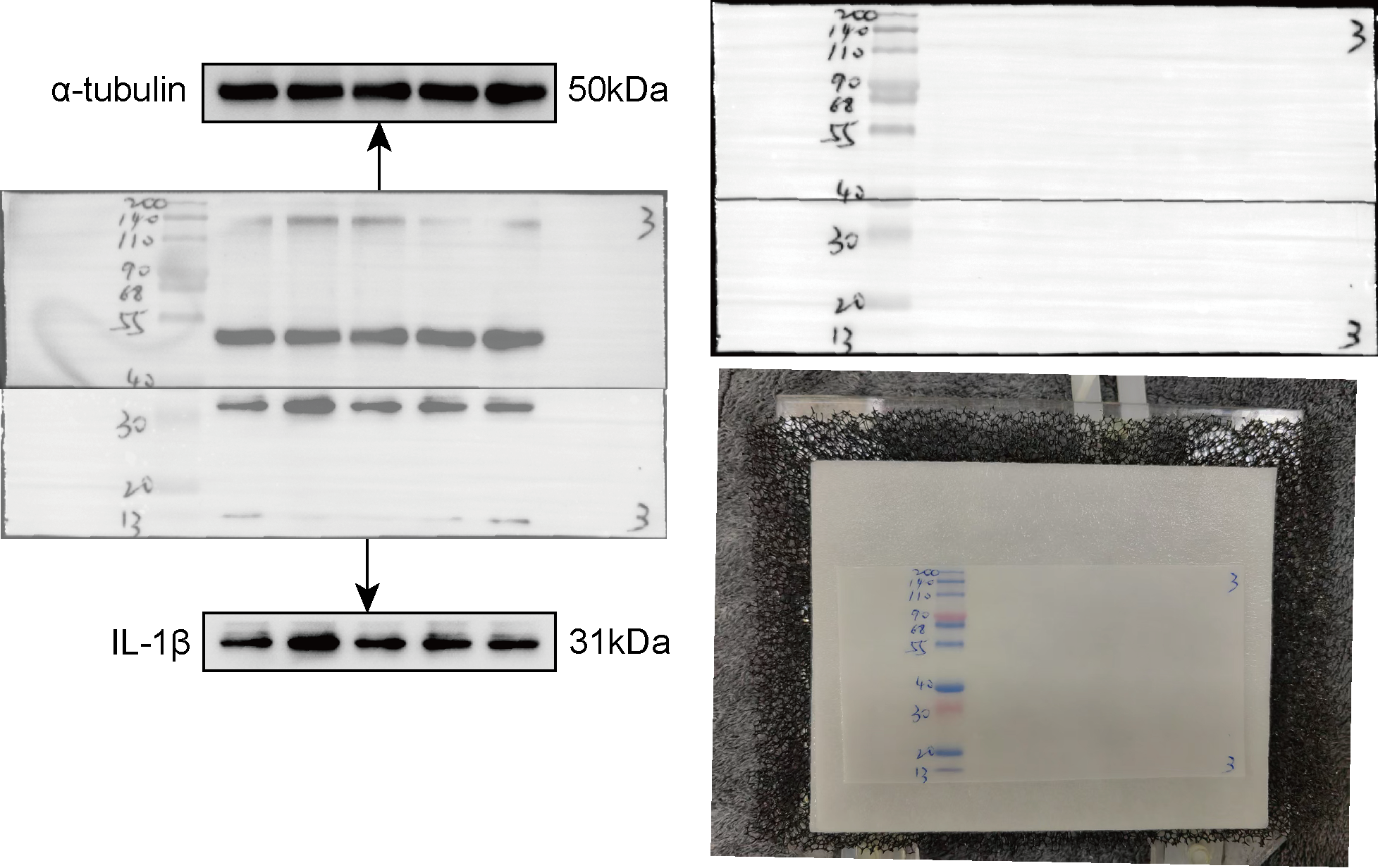

Supplement: Supplementary file 1 — Supplementary Material 1 [file 12993_2025_282_MOESM1_ESM.zip › WB figure/IL-1β.tif]

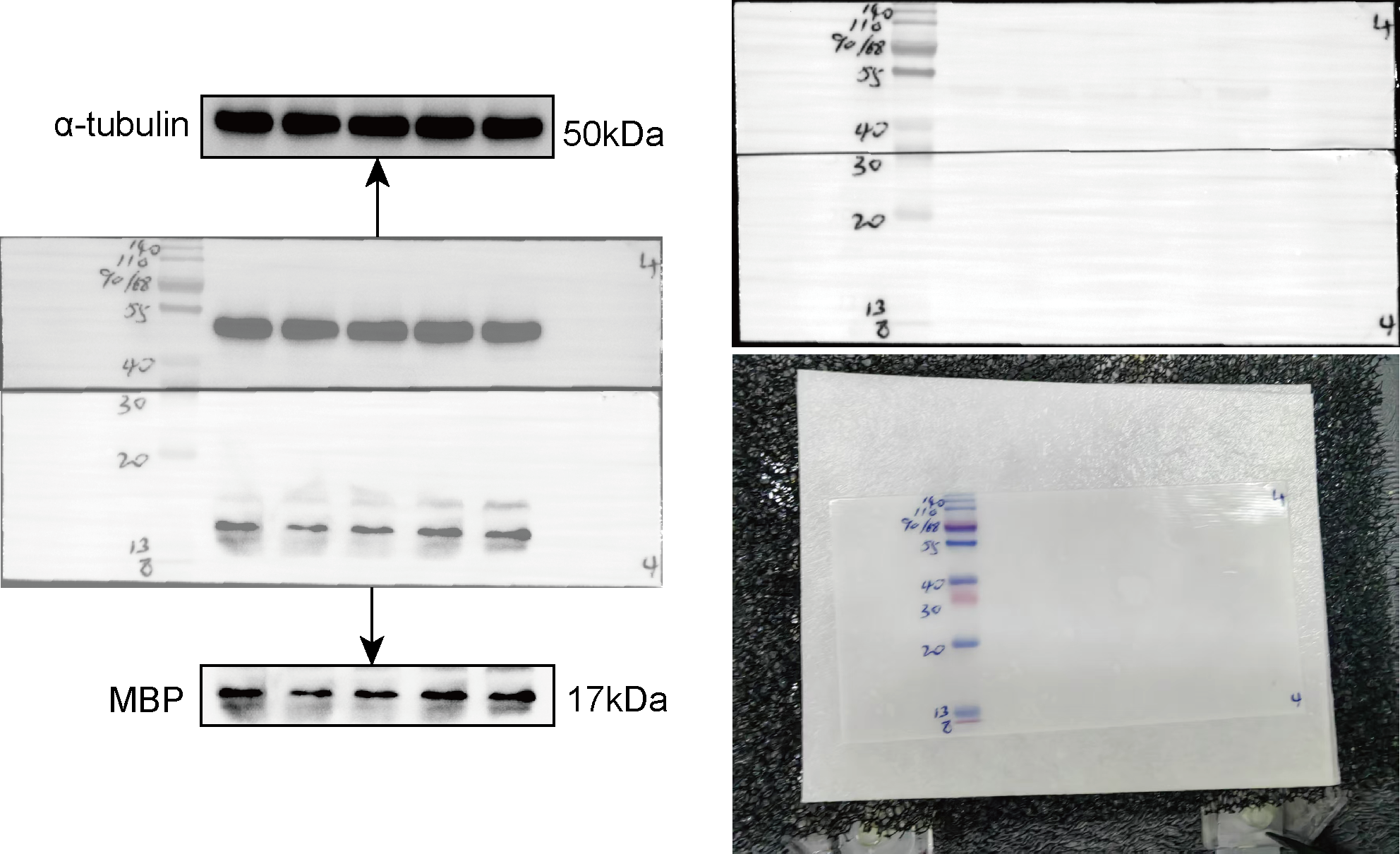

Supplement: Supplementary file 1 — Supplementary Material 1 [file 12993_2025_282_MOESM1_ESM.zip › WB figure/MBP.tif]
